# Mechanism of Electrochemical Deposition and Coloration of Electrochromic V_2_O_5_ Nano Thin Films: an In Situ X-Ray Spectroscopy Study

**DOI:** 10.1186/s11671-015-1095-9

**Published:** 2015-10-05

**Authors:** Ying-Rui Lu, Tzung-Zing Wu, Chi-Liang Chen, Da-Hau Wei, Jeng-Lung Chen, Wu-Ching Chou, Chung-Li Dong

**Affiliations:** Department of Physics, Tamkang University, New Taipei, 25137 Taiwan; Program for Science and Technology of Accelerator Light Source, National Chiao Tung University, Hsinchu, 30010 Taiwan; National Synchrotron Radiation Research Center (NSRRC), Hsinchu, 30076 Taiwan; Department of Mechanical Engineering and Institute of Manufacturing Technology, National Taipei University of Technology, Taipei, 106 Taiwan; Department of Electrophysics, National Chiao Tung University, Hsinchu, 30010 Taiwan

**Keywords:** In situ X-ray spectroscopy study, Electrochromism, V_2_O_5_ thin films

## Abstract

Electrochromic switching devices have elicited considerable attention because these thin films are among the most promising materials for energy-saving applications. The vanadium oxide system is simple and inexpensive because only a single-layer film of this material is sufficient for coloration. Vanadium dioxide thin films are fabricated by electrochemical deposition and cyclic voltammetry. Chronoamperometric analyses have indicated that the thin V_2_O_5_ film demonstrates faster intercalation and deintercalation of lithium ions than those of the thick V_2_O_5_ film, benefiting the coloration rate. Despite substantial research on the synthesis of vanadium oxides, the monitoring of electronic and atomic structures during growth and coloration of such material has not been thoroughly examined. In the present study, in situ X-ray absorption spectroscopy (XAS) is employed to determine the electronic and atomic structures of V_2_O_5_ thin films during electrochemical growth and then electrochromic coloration. In situ XAS results demonstrate the growth mechanism of the electrodeposited V_2_O_5_ thin film and suggest that its electrochromic performance strongly depends on the local atomic structure. This study improves our understanding of the electronic and atomic properties of the vanadium oxide system grown by electrochemical deposition and enhances the design of electrochromic materials for potential energy-saving applications.

## Background

The recent years have witnessed growing environmental concerns and increasing energy demands [1]. In the USA, up to 40 % energy is used for primary energy consumption of buildings, which contribute over 30 % CO_2_ emissions [2]. Thus, the effective use of energy has increasingly become an important issue. Smart windows can change optical properties by using an applied electric field or current, thereby avoiding excessive solar heating while taking advantage of heating mechanisms when necessary. Vanadium oxide systems comprise many oxide phases, including VO, V_2_O_3_, VO_2_, V_6_O_13_, V_3_O_7_, and V_2_O_5_. In particular, vanadium pentoxide is the most stable oxide in such systems. V_2_O_5_ exhibits an energy gap of approximately 2.2 eV and undergoes semiconductor–metal transition at around 250 °C. V_2_O_5_ materials demonstrate a color change when lithium ions are injected into or extracted from the layer spaces. Hence, V_2_O_5_ materials provide significant potential for applications in energy-saving devices, such as catalysts, sensors, electronic materials, and battery electrodes [3–5]. V_2_O_5_ films have been fabricated by various methods, such as reactive DC magnetron sputtering [6], vacuum-evaporated deposition [7], chemical vapor deposition [8], sol–gel method [9], spray pyrolysis technique [10], and electrodeposition [11]. Electrochemical method is greatly advantageous over other methods in terms of economics and flexibility; typically, this method can also be used to fabricate mesostructured thin films [12]. The high surface area of the mesoporous structure of V_2_O_5_ thin films provides porous channels, which facilitate fast ion diffusion and effective strain relaxation upon cycling of Li ion intercalation and deintercalation. Numerous studies on the application of V_2_O_5_ in energy-saving (smart windows [13]) or energy storage devices (batteries/supercapacitors [14]) have reported various synthesis methods [15], electrochemical properties, and photocatalytic performances [16]. Several studies have also demonstrated the importance of electronic structure characterization [17–19]. Eyert et al. [17] indicated that octahedral distortions enhance the optical band gap. Willinger et al. [18] compared the basic structural VO_5_ units in α-V_2_O_5_ and γ-V_2_O_5_ with regard to the differences in their geometric and electronic structures. The relation between geometric and electronic structures is critical because the basic structural unit is also common to industrial vanadium phosphorus oxide (VPO) catalysts [18]. However, the detail determination of the local atomic/electronic structure during growth of such electrochemically deposited film and during electrochromic coloration has not been thoroughly examined because of the lack of proper characterization tools. In the current study, an in situ electrochemical liquid cell was built, and in situ synchrotron hard X-ray absorption spectroscopy (XAS) was performed to monitor the electronic and atomic structures during the electrochemical deposition of V_2_O_5_ nano thin films. Changes in atomic/electronic structure upon coloration were determined. This approach is critical in determining the electrochemical growth and coloration mechanism, providing a great opportunity to further understand and improve the electrochemical properties of electrochromic materials.

## Methods

### Characterization and Measurement of Electrochemical Properties

Scanning electron microscopy (SEM, model PHILIPS S-4300) was used to examine the surface morphology. Electrodeposition was performed on a potentiostat/galvanostat (Princeton Applied Research, Versa STAT 4). A conventional three-electrode cell was utilized with an ITO substrate. V_2_O_5_ thin films were electrodeposited to a 1:1 mixture of deionized water and ethanol, which contained 1 M VOSO_4_·xH_2_O. Electrodepositions were performed at −0.7 V against the reference electrode for 20, 40, and 60 s. The lithium-ion intercalation/deintercalation properties of the V_2_O_5_ material were investigated in 1 M LiClO_4_ solution containing propylene carbonate. Cyclic voltammetry was performed between −1 and +1.25 V (versus Pt) at a scanning rate of 0.025 V s^−1^ using a potentiostat/galvanostat (Princeton Applied Research, Versa STAT 4). Chronoamperometric measurements were obtained at a constant voltage of +0.5 V, and the current change was monitored for 45 s at room temperature.

### Measurement of Electronic Structure

**Fig. 1:**
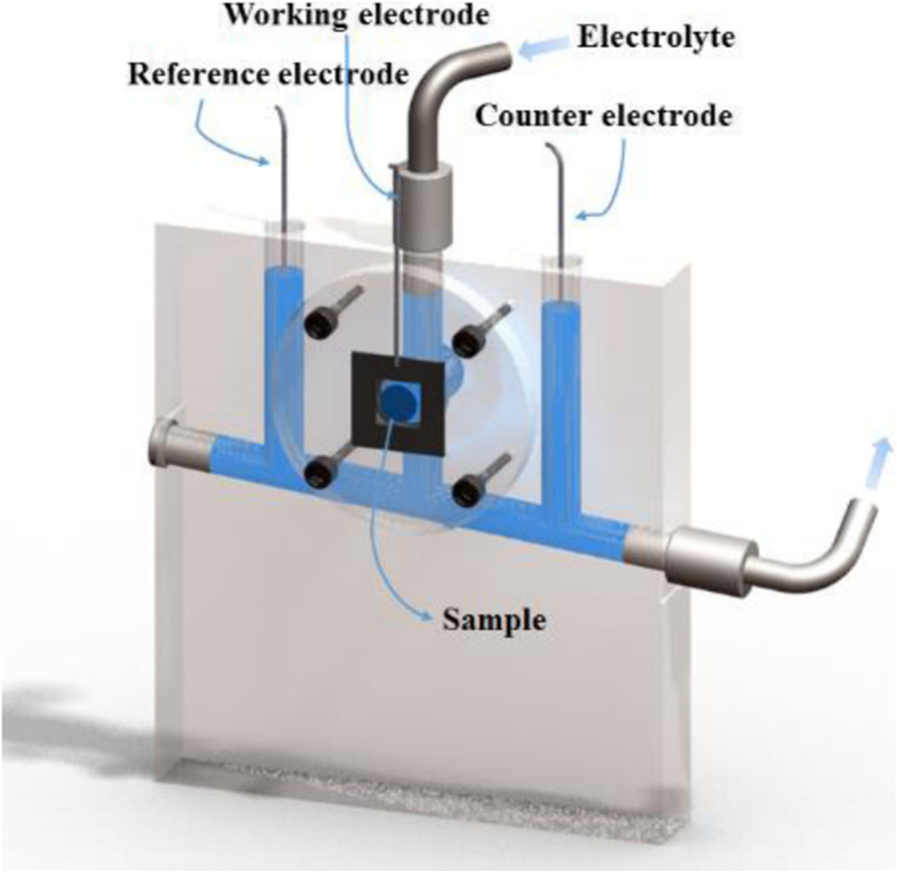
Schematic of electrochemical cell assembly for in situ XAS studies

V K-edge XAS spectra were collected at the hard X-ray beamline BL17C of the NSRRC. Electrodeposition of the V_2_O_5_ electrodes was performed potentiostatically at 0.7 V at room temperature using Au-coated Si_3_N_4_ as the substrate in the in situ reaction cell. Pt electrodes were used as the counter and reference electrodes. Figure 1 presents a schematic of the substrate-film-sample holder assembly in contact with the VOSO_4_·xH_2_O and LiClO_4_ electrolyte. The total fluorescence yield signal was collected with this sample holder. The samples were deposited onto a Si_3_N_4_ membrane window and used as working electrodes in a three-electrode setup. Prior to the deposition of the V_2_O_5_ films, 10-nm Au layers were evaporated on the Si_3_N_4_ window to provide an adhesive metallic current collector between V_2_O_5_ and Si_3_N_4_. The Si_3_N_4_ membrane window is transparent for X-rays; the membrane is attached to a PVC supporting frame by an Araldite® adhesive that creates a tight seal.

## Results and Discussion

**Fig. 2:**
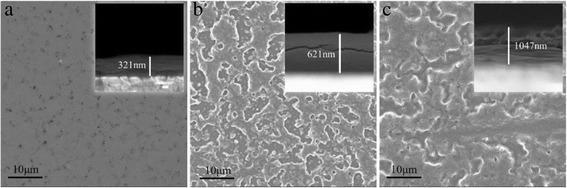
SEM image of V_2_O_5_ electrode deposited for **a** 20, **b** 40, and **c** 60 s

Figure 2 shows SEM which examines the surface morphology of the electrochemical growth of the V_2_O_5_ thin films. The V_2_O_5_ thin films that were deposited for 20 s were relatively smooth and adhered very well onto the substrate, as shown in Fig. 2a. As the deposition time increased to 40 and 60 s, the morphology of the V_2_O_5_ thin films significantly changed (Fig. 2b, c). The morphology of the V_2_O_5_ thin films changed from smooth to typical sea-island morphology. The film thickness of the electrochemical growth of the V_2_O_5_ thin films was measured using the cross-sectional SEM images (inset of Fig. 2a–c), with thicknesses of 321, 621, and 1047 nm for the deposition times of 20, 40, and 60 s, respectively.

**Fig. 3:**
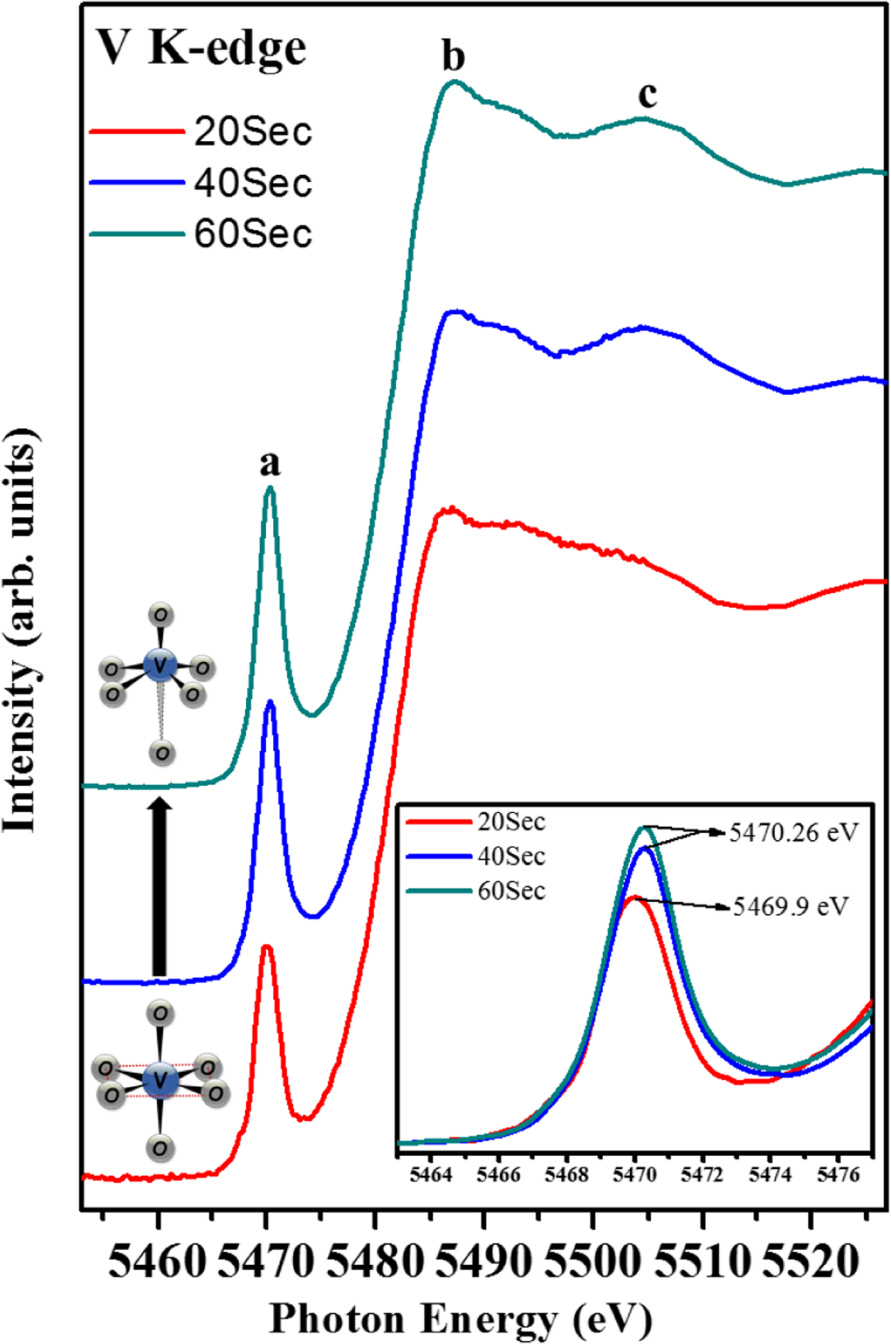
Vanadium K-edge XAS spectra of V_2_O_5_ electrodes deposited for 20, 40, and 60 s. The *inset* magnifies the pre-edge region

In situ K-edge XAS was employed to investigate the electrochemical growth of the V_2_O_5_ thin films in both local electronic and atomic structures as a function of time. Figure 3 shows a comparison of the normalized V K-edge spectra for the V_2_O_5_ films deposited at different times (20, 40, and 60 s). Numerous reports dealing with XAS spectra of V_2_O_5_ have already been published. The first feature shown in the spectra is the pre-edge peak (a). The intensity and energy position of the pre-edge may be used qualitatively to derive structural and chemical information. Pre-edge absorption is associated with dipole forbidden *s* → *d* transitions, which become allowed in the vanadium 3D states mixed with oxygen *p* states arising from the noncentrosymmetric environment of the slightly distorted octahedral [20]. Hence, the pre-edge peak intensity is very sensitive to alterations in the local geometrical symmetry [21]. Features b and c originate from the 1*s* core-electron excitation to the 4*p* orbital. The above features are highly sensitive to the effective valence state of the vanadium and the chemical environment surrounding the vanadium site [22, 23]. The V_2_O_5_ thin films deposited for 20 s are most likely in the form of V^5+^ with octahedral symmetry [19, 23]. Examination of the spectra with deposition time reveals that the edge energy shifted to increased values (inset of Fig. 3a), suggesting that vanadium is most likely in the form of V^5+^ with pyramid symmetry in the V_2_O_5_ electrode deposited for 40 and 60 s [19, 24]. In addition to the shift in edge energy, the intensity of the pre-edge peak also increases, suggesting an increase in the distortion of the VO_5_ square pyramid with increasing deposition time [25].

**Fig. 4:**
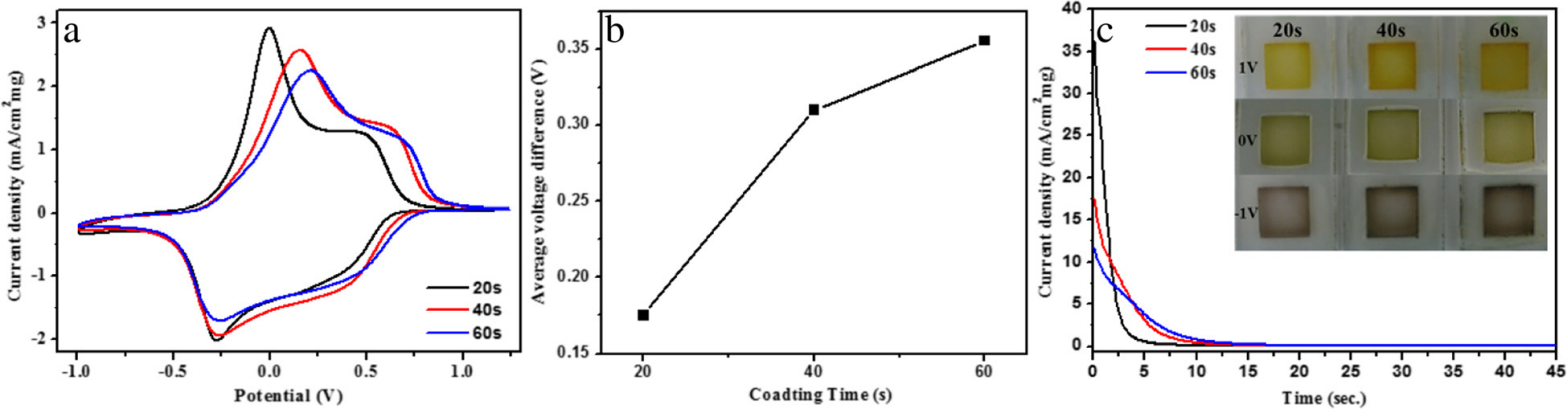
**a** Cyclic voltammograms of electrodeposited vanadium oxide films for lithium intercalation/deintercalation. **b** The average voltage difference between the cathodic and the corresponding anodic double peak of the redox reactions. **c** Chronoamperometry of vanadium oxide films

The electrochemical behavior of the V_2_O_5_ material was investigated using the three-electrode system, in which Pt foils were used as the counter and reference electrodes. Cyclic voltammetry and chronoamperometry were adopted to investigate the Li insertion/extraction behavior in 1 M LiClO_4_ propylene carbonate solution with a scan rate of 0.25 V s^−1^. Figure 4a shows typical cyclic voltammograms of V_2_O_5_ electrodes with different deposition times in the potential range of −1 to 1.25 V. The electrochemical Li^+^ insertion process occurring at V_2_O_5_ electrodes can be expressed by V_2_O_5_ + x Li^+^ + xe^−^ ↔ Li_x_V_2_O_5_ [14, 26], which is accompanied by color changes. These processes lead to film coloration [yellow → green → blue (inset of Fig. 4c)]. The V_2_O_5_ electrode shows two sets of broad, symmetric, and well-separated redox peaks, indicating sluggish lithium ion insertion/deinsertion kinetics. Moreover, compared with the V_2_O_5_ electrode at 40 and 60 s deposition times, all the peaks from the V_2_O_5_ electrode at 20 s deposition time shifted to decreased potentials (Fig. 4a). The average voltage difference between the cathodic and the corresponding anodic double peaks of the redox reactions (Fig. 4b) decreases from 350 mV at 60 s deposition time to 175 mV at 20 s deposition time, indicating easier intercalation and deintercalation of lithium ions. To compare the coloration efficiencies directly, the V_2_O_5_ electrodes at different deposition times were first biased at −0.5 V versus a Pt reference electrode for 45 s to facilitate lithium intercalation. The polarity was then switched immediately to +0.5 V to initiate lithium deintercalation, recording the change in current (Fig. 4b). Compared with the V_2_O_5_ electrode deposited for 20 s, the V_2_O_5_ electrode deposited for 40 and 60 s showed a very slow current decay, indicating that the charge characteristics, such as deposition times, are much slower in cation deintercalation. In the case of the V_2_O_5_ electrode deposited for 20 s, the initial increase in current was much higher than that of the V_2_O_5_ electrodes deposited for 40 and 60 s. This finding implies that the V_2_O_5_ electrode deposited for 20 s indicates faster intercalation and deintercalation of lithium ions. The higher kinetics of lithium insertion/extraction in the V_2_O_5_ electrode deposited for 20 s depends not only on the film thickness of the electrode but also on the electronic and atomic structures, as revealed in greater detail by the in situ K-edge XAS discussed later.

**Fig. 5:**
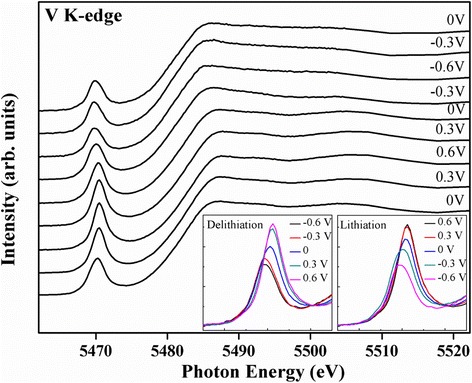
In situ XAS at V K-edge for 60 s at applied potentials in the order of 0.0, 0.3, 0.6, 0.3, 0, −0.3, −0.6, −0.3, and 0 V. *Insets* display the enlargements of pre-peak intensities during delithiation (*left*) and lithiation (*right*)

In situ XAS at V K-edge was performed during the coloration process upon the electrochemical reaction to gain insights into the effects of delithiation/lithiation on the oxidation states of vanadium, as well as the local atomic structure of the V_2_O_5_ thin films. Although the thinner film (deposited for 20 s) exhibits faster coloration rate than a thicker film (deposited for 60 s), the thicker film is more stable than the thinner one. Thus, the XAS spectrum of a V_2_O_5_ film deposited for 60 s is shown in Fig. 5. The insets compare the pre-peak intensities during delithiation (left panel) and lithiation (right panel) processes. Notably, two major changes under bias potential from 0 to −0.6 V caused by Li-ion intercalation in the spectral features, which were observed in the pre-edge region, were indicated as follows: a shift of pre-edge peak position to the lower energy and a decrease in pre-edge peaks. The shift to low energy was attributed to the decreased oxidation state of metal ions because of Li-ion intercalation, which can be conceptually caused by the reduced effective nuclear charge of the metal ions with decreased oxidation state. The pre-edge peak intensity clearly decreased for the bias potentials from 0 to −0.6 V, suggesting that the local structure around the V atom became more symmetrical with Li-ion intercalation. However, extracting lithium under a bias potential from −0.6 to +0.6 V changes the color of the film from deep blue to green to yellow. At −0.3, 0, and 0.6 V, the pre-edge peak of the V K-edge shifts to high energy when the film was oxidized, and partial V^4+^ ions change into V^5+^. The remaining V^4+^ ions reduced to V^5+^ at 0.6 V. The positive shift in the V K-edge indicates that the average oxidation state of V increased because of an increase in the attractive potential of the nucleus. Additionally, the increase in the pre-edge peak area corresponds to the gradually decreasing lithium content, revealing that structural symmetry was modified from O_h_ (V^4+^) to a mixture of P_y_ (V^5+^). Without the in situ electrochemical cell, the above atomic/electronic structure information cannot be obtained. Overall, these results emphasize the importance of in situ X-ray spectroscopic characterization on the atomic/electronic structures of energy materials in their working condition.

## Conclusions

Electrochromic switching devices have been widely investigated because such switching can control the throughput of visible light and solar radiation into buildings by applying electrical voltage, thereby imparting energy efficiency. Electrochemical in situ XAS studies indicated that the electrodeposition of V_2_O_5_ under constant 0.7 V conditions produced V_2_O_5_ thin films with different local electronic and atomic structures along with variation of film thickness. Given the increasing film thickness, the local geometrical symmetry of V_2_O_5_ thin films varies from V^5+^ with octahedral symmetry to V^5+^ with pyramid symmetry. In situ XAS was performed in the present study to monitor the effects of delithiation/lithiation on the vanadium oxidation states and the local atomic structures of the V_2_O_5_ thin films. Color switching upon the intercalation of hydrogen is caused by the valence change of the cations. Such effect is accompanied by structural rearrangement. This in situ XAS electrochemical cell setup allows real-time monitoring of the element-specific electronic structural changes in a system at all stages of electrochemical reaction. It is anticipated that using this technique, the growth parameters of V_2_O_5_ thin films can be fine-tuned, achieving optimization.
